# Phenotypic Correlates of Structural and Functional Protein Impairments Resultant from ALDH5A1 Variants

**DOI:** 10.21203/rs.3.rs-3111263/v1

**Published:** 2023-07-10

**Authors:** Itay Tokatly Latzer, Jean-Baptiste Roullet, Samuele Cesaro, Melissa L. DiBacco, Erland Arning, Alexander Rotenberg, Henry H C Lee, Thomas Opladen, Kathrin Jeltsch, Àngels García-Cazorla, Natalia Juliá-Palacios, K. Michael Gibson, Mariarita Bertoldi, Phillip L. Pearl

**Affiliations:** Boston Children’s Hospital, Harvard Medical School; Washington State University; University of Verona; Boston Children’s Hospital, Harvard Medical School; Baylor Scott & White Research Institute; Boston Children’s Hospital, Harvard Medical School; Boston Children’s Hospital, Harvard Medical School; University Children’s Hospital Heidelberg; University Children’s Hospital Heidelberg; Institut de Recerca, Hospital Sant Joan de Déu; Institut de Recerca, Hospital Sant Joan de Déu; Washington State University; University of Verona; Boston Children’s Hospital, Harvard Medical School

**Keywords:** SSADH Deficiency, 4-hydroxybutyricuria, variants, genotype-phenotype, in-silico

## Abstract

**Objective:**

To investigate the genotype-to-protein-to-phenotype correlations of succinic semialdehyde dehydrogenase deficiency (SSADHD), an inherited metabolic disorder of γ-aminobutyric acid catabolism.

**Methods:**

Bioinformatics and in silico mutagenesis analyses of ALDH5A1 variants were performed to evaluate their impact on protein stability, active site and co-factor binding domains, splicing, and homotetramer formation. Protein abnormalities were then correlated with a validated disease-specific clinical severity score and neurological, neuropsychological, biochemical, neuroimaging, and neurophysiological metrics.

**Results:**

A total of 58 individuals (1:1 male/female ratio) were affected by 32 ALDH5A1 pathogenic variants, eight of which were novel. Compared to individuals with single homotetrameric or multiple homo and heterotetrameric proteins, those predicted not to synthesize any functional enzyme protein had significantly lower expression of *ALDH5A1* (*p* = 0.001), worse overall clinical outcomes (*p* = 0.008) and specifically more severe cognitive deficits (*p* = 0.01), epilepsy (*p* = 0.04) and psychiatric morbidity (*p* = 0.04). Compared to individuals with predictions of having no protein or a protein impaired in catalytic functions, subjects whose proteins were predicted to be impaired in stability, folding, or oligomerization had a better overall clinical outcome (*p* = 0.02) and adaptive skills (*p* = 0.04).

**Conclusions:**

The quantity and type of enzyme proteins (no protein, single homotetramers, or multiple homo and heterotetramers), as well as their structural and functional impairments (catalytic or stability, folding, or oligomerization), contribute to phenotype severity in SSADHD. These findings are valuable for assessment of disease prognosis and management, including patient selection for gene replacement therapy. Furthermore, they provide a roadmap to determine genotype-to-protein-to-phenotype relationships in other autosomal recessive disorders.

## Introduction

Succinic semialdehyde dehydrogenase deficiency (SSADHD) (OMIM #271980) is a rare (prevalent in ~ 1/460,000^1^) inherited metabolic disorder caused by autosomal recessive inheritance of *ALDH5A1* sequence variants^2^. Enzyme deficiency results in impaired γ-aminobutyric acid (GABA) catabolism and its accumulation along with other GABA-related metabolites such as guanidinobutyrate (GBA) and γ-hydroxybutyrate (GHB). The phenotype ranges in the severity of a broad spectrum of non-pathognomonic symptoms (cognitive, adaptive, and communication deficits, movement disorders, seizures, sleep disturbances, and psychiatric manifestations such as inattention, hyperactivity, and obsessive-compulsive behaviors^3^). Attempts to develop gene therapy for SSADHD are ongoing^4^, but current treatment options remain supportive.

*ALDH5A1* spans > 38kB on chromosome 6p22 and has an open reading frame of 1605 base pairs that encodes 535 amino acids. Its resultant protein, SSADH, is a mitochondrial enzyme composed of identical monomers arranged in a tetrameric quaternary structure. The crystal structure of human SSADH shows that each monomer comprises an NAD^+^ binding domain (amino acids 1–173, 196–307, and 509–524), a catalytic domain (amino acids 308–508), and an oligomerization domain (amino acids 174–195 and 525–535)^5^. Sixteen identified missense mutations determine the substitution of amino acids in different protein domains, impacting both its structure and function. For six of these missense mutations, a preliminary prediction of their amino acid alteration consequence has been proposed based on the combination of visualization of the affected residues’ position in the crystal structure^5^ and data of activity cell-free extracts^5, 6^.

A lack of a clear correlation between genotype to disease-specific phenotype^7^ hinders our ability to define disease management criteria, offer definite prognostic counseling, and develop novel therapies for SSADHD. This study, which includes the largest cohort of genetically confirmed SSADHD subjects, aimed first to investigate how *ALDH5A1* sequence variants structurally and functionally impact the enzyme SSADH. This was accomplished by *in silico* mutagenesis and in-depth bioinformatic analyses of the chemical and network effects resulting from the substitution of each SSADH residue. The results of these analyses yielded subgroups of quantitative, structural, and functional SSADH molecular impairments. The study’s second aim was to correlate between these subgroups to outcomes of clinical, neurophysiological, biochemical, and neuroimaging assessments representing the clinical phenotype of SSADHD.

## Methods

### Settings and population

This study presents an analysis of data gathered from a natural history study of SSADHD (ClinicalTrials.gov ID: NCT03758521; Boston Children’s Hospital Institutional Review Board #P00029917), a prospective and multinational study commenced in 2018 by investigators of the SSADH Deficiency Research Consortium and funded by a grant to Washington State University from the National Institutes of Health (NIH R01 1R01HD091142). Clinical assessments and specimen collections were performed at three main clinical sites [Boston Children’s Hospital (BCH) in the United States, University Children’s Hospital Heidelberg (UCHH) in Germany, and Hospital Sant Joan Déu Barcelona Children’s Hospital (UDB) in Spain] with the University of Florida providing data management. In silico mutagenesis and bioanalytical variant analyses were performed by collaborators at the University of Verona, Italy.

After being genetically confirmed with SSADHD, subjects enrolled in the natural history study undergo a series of clinical and laboratory assessments biennially. Disease severity is measured using a validated clinical severity score (CSS) obtained at bedside during study visits^8^. The CSS is a composite score obtained by scoring the severity of five domains representing the main manifestations of SSADHD: cognitive function, communication, motor function, psychiatric manifestations, and epilepsy. Each domain is scored on a 1–5 scale (1 indicating the most severe clinical phenotype). Participants also undergo neuropsychological evaluations, magnetic resonance imaging (MRI) and magnetic resonance spectroscopy (MRS), electroencephalography (EEG), blood collection for GABA, GHB, and γ-guanidinobutyrate (GBA), and transcranial magnetic stimulation (TMS) studies, the latter which are only completed at the BCH site.

### Bioinformatics analyses assessing missense, deletion, or insertion mutations

The functionality of SSADH variants was determined using web-available bioinformatics tools based on the variants’ effect on polypeptide chains (truncation or amino acid substitution). Scale-invariant feature transform (SIFT) predicted whether the variant is deleterious or tolerated, and Polymorphism Phenotyping v2 (POLYPHEN2) scored the variant as probably or possibly damaging. Gibbs free energy change (ΔΔG, kcal/mol) was used to assess the stability of SSADH variants (destabilizing unfavorable, destabilizing favorable, stabilizing favorable) and determined using CUPSAT 9. These methods refer to the single polypeptide chain and do not consider the fact that SSADH is a functional oligomer assembled by four monomeric polypeptide chains 5. This resulted in different possible SSADH polypeptide chain combinations based on the patient’s genotype 10. Homozygotic missense mutations are presumed to lead to the same amino acid alteration and synthesize the same SSADH polypeptide. Compound heterozygotes are predicted to synthesize two SSADH polypeptide chains, each with a different amino acid change. The theoretical random combination of these polypeptide chains leads to different homo and heterotetrameric species. If the genetic variant (insertion or deletion) results in a premature stop codon on both alleles, a functional SSADH protein would not be synthesized. However, in compound heterozygotes, it may lead to only one functional homotetrameric SSADH 10. BindProfX was used to predict changes in the binding affinity of monomers to form dimers or tetramers upon variation in the form of ΔΔG values, based on an algorithm that combines FoldX physics-based potentials with conservation scores from pairs of protein-protein interaction surface sequence profiles. Conservation analyses were performed with the Consurf Server (https://consurf.tau.ac.il/) using the human SSADH amino acidic sequence as input data. A conservation score from 1 (variable residue) to 9 (conserved residue) has been attributed to each residue. Standard molecular diagnostic mutation nomenclature was followed 11.

### In silico analysis of amino acid substitutions in SSADH protein variants

Structural analysis and *in silico* mutagenesis of the three-dimensional structure of human SSADH (PDB: 2W8N) solved by Kim and colleagues^5^ was carried out by PyMOL Molecular Graphics System (version 2.5.2, Schrödinger LLC.). The types of substitution, residue localization, microenvironment, and interactions were analyzed. The lower strain value expressed by the software from the *in silico* mutagenesis was exploited to choose the more stable rotamer and as an informative factor for steric hindrance caused by the substitution of the wild-type amino acid residue with the amino acid substituted as a result of the patient’s missense mutation. Molecular interactions between residues were identified in a surrounding area of 5 Å by means of the web tool Mol* Viewer 12.

### Splice site analysis of intronic variants

Analysis of splice-site variants was performed using SpliceAI^13^, an interface for splicing prediction ideal for intronic variants that are +/− 50 bp from exon borders. The Δ score achieved by this method for each splice-site variant ranges from 0 to 1 and can be interpreted as the probability that the variant affects splicing at any position within a window around it (+/− 50 bp by default)^14^. A Δ cut-off score > 0.5 confidently denotes aberrant splicing has occurred; scores > 0.8 indicate the prediction is highly precise; scores ranging from 0.2 to 0.5 assume the occurrence of alternative splicing with production of an aberrant and normal transcript; and scores < 0.2 imply the variant had no effect.

### Clinical and neurophysiological assessments

#### Neuropsychological assessments

Neuropsychological tests evaluated cognitive and adaptive skills (Mullen Scales of Early Learning^15^; Differential Abilities Scale, 2nd Edition^16^; Wechsler Abbreviated Scales of Intelligence^17^; Vineland Adaptive Behavior Scales, 2nd Edition^18^), language capacity (Receptive Language and Expressive Language, REEL-3^19^), primary communication (Autism Diagnostic Observation Scale-2, ADOS-2^20^), motor function (Movement Assessment Battery for Children- 2nd Edition^21^), and psychiatric and behavioral attributes [Achenbach Child or Adult Behavior Checklist (CBCL or ABCL, respectively)^22–24^].

#### Magnetic Resonance Imaging and Spectroscopy

MRIs are done by a whole-body 3-Tesla MRI scanner (Siemens Skyra, Erlangen, Germany). Estimations of GABA/N-acetylaspartate (NAA) ratios result from two acquisitions (TR 1500ms; TE 68ms; bandwidth 1200Hz) of GABA-specific MEGA-PRESS sequences of a single voxel sized 27cm^3^ (30mm×30mm×30mm). The first acquisition includes a 1.9 ppm-arranged editing pulse allowing selective refocusing of the GABA multiplet at 3.0 ppm, and a second acquisition allocates the inversion from another location, enabling to determine GABA’s J-evolution. The basal ganglia region is sampled since in SSADHD, MRI abnormalities were most consistently detected in this area^25^. The posterior cingulate and occipital cortices are sampled for their reliability to determine GABA measurements^26–28^. Spectroscopy data are processed by the LCModel9 software (version 6.3)^29^.

#### Electroencephalography

EEGs are done as 21-channel digital studies (Natus^®^ NeuroWorks^®^ EEG Software, Natus Medical Incorporated, Ontario, Canada) lasting ~ 30 minutes, with electrodes placed according to the 10/20 International System capture awake and sleep states. Parameters of bipolar and referential electrode montages consist of a 512Hz sampling rate and 24-bit analog-to-digital conversion.

#### Transcranial magnetic stimulation

TMS is applied using the Nexstim 5.1.1 system (Nexstim, Finland). Each participant’s anatomical T1-weighted MRI sequence is used for co-registration and frameless stereotaxy. The primary motor cortex is detected using a figure-of-eight coil, while motor-evoked potentials are recorded from the contralateral abductor pollicis brevis (APB) muscle^30^. Electromyography (EMG) is recorded at 3 kHz using a bandpass filter ranging between 10–500 Hz. Resting motor threshold (rMT) is defined as the operational minimum machine output required to elicit a motor-evoked potential ≥50 μV from the target resting muscle in >50% of trials. Cortical silent period (CSP) is defined as the duration from stimulation at 150% rMT to the spontaneous return of voluntary EMG-detected muscle activity on the target muscle. Long-interval cortical inhibition (LICI), defined as the log transformation of the peak-to-peak amplitude of the second pulse’s resultant MEP from the peak-to-peak amplitude of the first pulse’s resultant MEP, was estimated by pairs of stimulations delivered at 120% rMT with interpulse intervals lasting 100 milliseconds. Analysis of EMG signals was performed via LabChart v8.1.17 to extract the CSP and LICI metrics.

#### Plasma GABA and GABA-related gene expression

Plasma GABA, GHB, and GBA concentrations were determined after hydrolysis with 6N HCl through stable isotope dilution liquid chromatography-mass spectrometry^31^. The expression of key GABA-related genes (ALDH5A1, Abat, Glud1, GLS) was determined in whole blood collected in PAXgene tubes. RNA extraction was performed using a PAXgene Blood miRNA Kit (QIAGEN, cat no. 763134, Hilden, Germany). RNA quality and concentration were determined using a Fragment Analyzer System Kit (cat. no. DNF-472–0500, Agilent, Santa Clara, CA) and the Qubit RNA HS assay kit (Invitrogen, cat. no. Q32855, Waltham, MA). cDNA was obtained using the RT2 First Strand Kit (QIAGEN, cat no. 330404) and loaded into a 384-well custom RT2 Profiler array (QIAGEN, Hilden, Germany). qPCR was performed using a CFX 384 (Bio-Rad Laboratories, Hercules, CA). Gene expression was normalized to GAPDH expression and expressed as 2−ΔCt.

#### Statistical analyses

Data were analyzed using SPSS Statistics (IBM SPSS Statistics, Version 28, 2021, IBM Corp, Armonk, NY, USA). Categorical variables are reported as their relative frequencies, whereas continuous variables are reported as either mean ± standard deviations (mean ± SD) or median and interquartile ranges (IQR) after testing for distribution normality. Group comparison and correlations were performed using either parametric tests (t test, one-way ANOVA, and Pearson correlation) or non-parametric tests (Mann-Whitney, Kruskal-Wallis, and Spearman’s rank correlation) as appropriate. Significance level thresholds of multiple comparisons were corrected by the Bonferroni-Dunn method. Relationships between genotypes and phenotypic variables known to be age-dependent (e.g., plasma GABA, GHB, and GBA, MRS-derived GABA/NAA ratio, and TMS-derived rMT and CSP) were analyzed using a linear regression model that included age as a covariate to obtain estimates of the differences between subgroups’ marginal means and their standard errors. A *p* value ≤ 0.05 was considered significant for all analyses.

## Results

**Figure 1:**
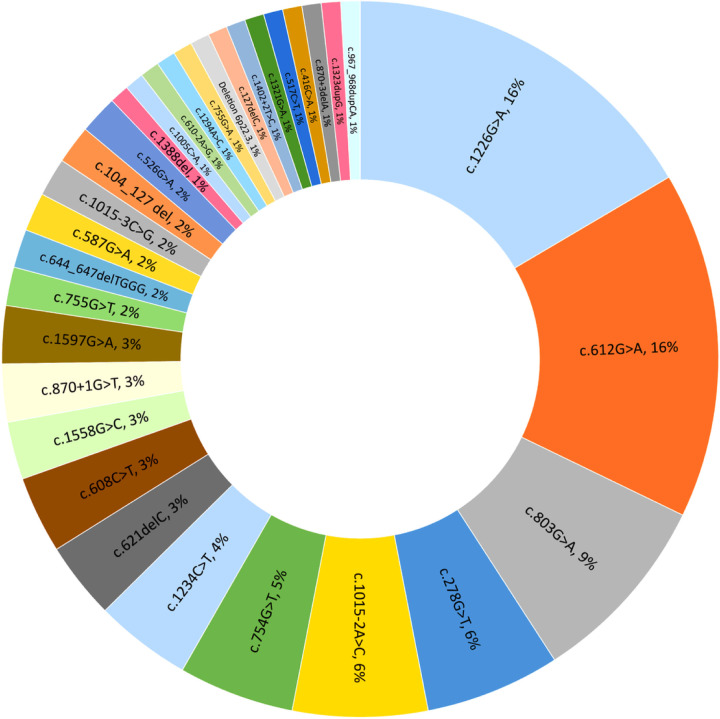
Rate of occurrence of 32 ALDH5A1 variants in 58 individuals with succinic semialdehyde dehydrogenase deficiency.

The study included the genetic information of 58 (1:1 male/female ratio) individuals from 50 unrelated families with 32 ALDH5A1 allelic variants, eight of which were previously unreported (c.104_127 del, c.127delC, c.870 + 1G > T, 6p22.3 deletion, c.644_647delTGGG, c.1558G > C, c.755G > A, and c.1388del) (Table 1). The most common sequence variants were c.1226G > A (16%), c.612G > A (16%), and c.803G > A (9%) (Fig. 1). Of the 116 affected alleles, 64 variants (55%) were missense, 23 non-sense (20%), 16 splice-site (14%), and 12 frameshift (10%) (10 deletions and 2 duplications). An additional variant included the deletion of chromosome 6p22.3 in its entirety. Twenty-three subjects (40%) were homozygotes, and 35 (60%) compound heterozygotes for these variants, resulting in either a truncated/no protein [N = 16 (28%)], a single homotetrameric protein with [N = 35 (60%)], or a mixed population of homo and heterotetrameric proteins [N = 7 (12%)]. A functional analysis based on the domain where the amino acid subjected to substitution is located and on the impact of the substitution on the binding strength of the affected intramolecular network determined that SSADH proteins were either impaired in stability, folding, and oligomerization [N = 29 (50%)] or in catalytic activity [N = 13 (22%)] (Table 2).

### In silico analyses

**Figure 2:**
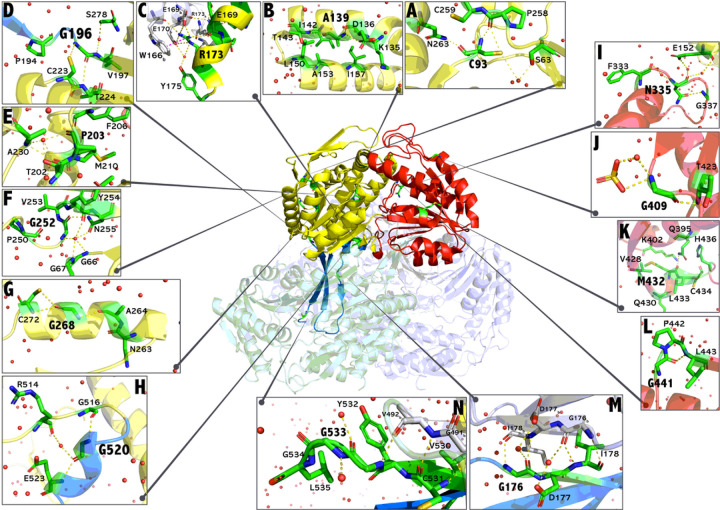
Representation of the SSADH amino acids subjected to substitution. Ribbon representation of the tetrameric assembly of human SSADH (PDB: 2W8N9) in which one monomer is colored by domain organization: NAD+ binding domain in yellow, catalytic domain in red, and oligomerization domain in blue. The other monomers are colored white, light green, and light purple. The amino acids that are subjected to substitution are represented by green sticks. A-H) Residues belonging to the NAD+ binding domain, I-L) residues belonging to the catalytic domain, and M-N) residues belonging to the oligomerization domain. For each residue, the main contacts with neighboring residues are displayed. The figure is rendered with PyMol (Molecular Graphics System (version 2.5.2, Schrödinger LLC).

According to the computational predictive tools POLYPHEN-2, SIFT, and CUPSTAT, all missense variants were determined to have a pathogenic clinical significance (Table 1) in accordance with the American College of Medical Genetics and Genomics (ACMG) standards and guidelines^32^. The variants’ functional consequences were investigated by studying the crystal structure of the SSADH enzyme. Initially, variants were mapped to the known domains of the protein: C93F, A139D, R173C, G196D, P203L, G252C, G252D, G252V, G268E, and G520R were mapped to the NAD^+^ binding domain; N335K, G409D, M432L, and G441R to the catalytic domain; and G176R and G533R to the oligomerization domain (Table 1, Fig. 2). Variants mapping to the NAD^+^ binding domain all lead to varying degrees of destabilizing or misfolding of the alpha/beta structure essential to the integrity of the NAD^+^ binding site. Since this domain is large, the functional effects of variants could vary depending on whether they lie on the surface of the NAD^+^ binding site, the NAD + binding groove, or in the secondary structures surrounding the site. This possibility led us to perform additional analyses of the spatial structure of each affected residue and refine our prediction of the effects of variants on protein function. C93 is present in a hydrophilic cluster (Fig. 2A), and the C93F substitution (c.278G > T) changes its interactions with nearby residues, leading to the collision of a bulky aromatic side chain with several structural elements and disruption of this domain’s stability. A139 lies within a hydrophobic interface between two antiparallel alpha-helices of the same monomer, with carbonyl and amidic moieties providing stabilizing polar interactions. The A139D substitution (c.416C > A) destroys the hydrophobic interface, leading to the disassembly of the alpha helices and domain destabilization (Fig. 2B). A173 is positioned at the edge of the NAD^+^ domain and is critical to maintaining the quaternary SSADH tetrameric structure assembled as a dimer of dimers^5^. A173 of one monomer faces the opposite monomer leading to the dimeric structure that will interact with an identical dimer to form the tetramer. The A173C substitution (c.517C > T) leads to the loss of interchain integrity while intrachain bonds remain stabilized by other means (Fig. 2C). G196 is involved in the linkage of two β sheets and is vital for stabilizing the entire NAD^+^ binding domain. The G196D substitution (c.587G > A) alters the stacking interactions of the two β-sheets (Fig. 2D) and destabilizes the NAD^+^ binding domain. P203, located in a buried residue within a hydrophobic cluster that accommodates the phosphate moiety of the coenzyme’s ADP portion, correctly positions the catalytic loop (residues 334–344). The P203L substitution (c.608C > T) alters the conformational rigidity of the residue and weakens the network bond (Fig. 2E). G252 resides in a loop connecting secondary structure elements responsible for the large eight stacked β-sheets composing the NAD^+^ domain, which is stabilized by an extensive H-bond network. When substitutions such as G252V (c.755G > T) (predicted to be the worst), G252C (c.754G > T), or G252D (c.755G > A) occur, polar and sterically bulkier residues are introduced into the hydrophobic moiety of the beta sheets and the fold of the eight-β-sheets element is compromised (Fig. 2F). G268 has a fundamental role in maintaining the stability of an α-helix essential for NAD^+^ binding. The G268E substitution (c.803G > A) loosens the structurally essential interactions of this region (Fig. 2G). Finally, G520 takes part in maintaining the secondary structure motif preceding the C-terminal by reinforcing an H-bondbackbone with other residues. Accordingly, the G520A substitution (c.1558G > C) leads to the disassembly of this region and deleterious protein misfolding (Fig. 2H).

The effects played by variants mapping at the catalytic domain are severe. N335K, by directly altering the catalytic loop, leads to larger functional than structural impairment. Alternatively, G409D, M432L, and G441R destabilize the architecture of the catalytic domain, resulting in its structural disassembly. In more depth, N335 belongs to the catalytic loop^5^ and maintains its orientation by means of an H-bond network. With the N335K substitution (c.1005C > A), the binding of succinic semialdehyde to its pocket is hindered (Fig. 2I). Since aspartate is a polar residue, the G409D substitution (c.1226G > A) destabilizes the superficial part of the β-sheet to which it belongs (Fig. 2J). The M432L replacement (c.1294A > C) alters the steric hindrance of this residue, possibly leading to erroneous rearrangement of nearby structures (Fig. 2K). Lastly, G441 belongs to a loop connecting structural elements of the catalytic domain, which is destabilized by the G441R substitution (c.1321G > A) (Fig. 2L).

Variants disturbing the oligomerization domain constitute two different Glycine-to-Arginine substitutions that preserve hydrophilicity but make interactions with the other monomer onerous despite mapping distantly on the protein surface. G176 maintains the H-bonds of nearby residues, and its substitution to arginine (c.526G > A) damages the multimeric assembly of the protein (Fig. 2M). The same holds for G533 residing on SSADH’s terminal loop, as its substitution by arginine (c.1597G > A) inhibits the proper stacking and inter-monomer interactions of this region (Fig. 2N).

Notably, 10/16 (62%) of the variants involve the substitution of a small glycine residue with a bulkier positively or negatively charged amino acid, resulting, at first glance, in a profound conformational effect, considering the conformational role played by glycine residues due to their relatively high degrees of freedom.

The steric hindrance resultant from neighboring residues within the monomeric structure of SSADH was highest in the variants G252V (67.95), G252C (56.81), G409D (56.27), G441R (55.84), and G252D (55.70) (Table 3), indicating a larger variation of their resultant protein from the wild-type protein. Interestingly, N335K and G176R variants have lower values of steric hindrance (Table 3) since the former affects the catalytic loop but does not play a steric effect, while the latter affects oligomerization with a neighboring monomer that is not reported by the steric hindrance calculation which is based on steric effects played on the same monomer.

### Splice-site variants analysis

Splice-site variant analysis with SpliceAI revealed that 5 out 6 of the splice-site variants (c.1015–2A > C, c.870 + 3delA, c.1402 + 2T > C, c.870 + 1G > T, c.610–2A > G) had high Δ scores (mean ± SD of 0.88 ± 0.12), indicating a high probability for aberrant splicing, alteration in the frame of the translated protein’s frame, and a predicted complete lack of SSADH protein. One splicing variant (c.1015–3C > G) resulted in a lower Δ score (0.39).

### Genotype-to-protein-to-phenotype correlations

Of the 58 study participants, 27 were assessed at BCH, 11 at UCHH, 10 at UDB, and 10 were from the SOC cohort. The study group had an even distribution of sexes, and participants’ overall median (IQR) age at their first study visit was 9.8 (5.4–15.3) years. The ethnic distribution was 40 (69%) Caucasians, 6 (10%) Arab, 3 (5%) Asian, and 9 (15%) other ethnicities. No participant was born prematurely or had any perinatal complications. Movement disorders were present in 32 (55%) subjects: 30 (52%) had ataxia, 14 (24%) had dyskinesia, and 9 (15%) had dystonia. Thirty subjects (52%) had seizures, which were considered drugresistant seizures^33^ in 9 (15%), and 47 (81%) had EEG abnormalities [27 (47%) with diffuse background slowing and 18 (31%) showing epileptiform activity]. The mean ± SD FSIQ was 51.0 ± 12.8 (assessed in 27 subjects), the total composite adaptive score was 60.5 ± 14.1 (assessed in 33 subjects), and 17/30 (57%) who were assessed with the ADOS-2 were diagnosed with ASD. The study group’s mean ± SD total CSS was 17.2 ± 2.8.

Compared to individuals with single homotetrameric or multiple homo and heterotetrameric proteins, those with no protein had significantly lower plasma expression of *ALDH5A1* (*p* = 0.001). They also had lower values of the total CSS (*p* = 0.008) and lower cognitive (*p* = 0.01), epilepsy (*p* = 0.04), and psychiatric (*p* = 0.04) severity scores. Dyskinesia (*p* = 0.05), seizures (*p* = 0.01), and EEG abnormalities (*p* < 0.001) were significantly more prevalent in individuals with no protein or single homotetramers compared to those with a mixed population of homo and heterotetrameric proteins. There was no significant relationship between the number of proteins and age, sex, communication and motor CSS domain scores, FSIQ, adaptive function, Autism Spectrum Disorder, and age-adjusted cerebral GABA/NAA ratio, plasma GABA, GHB, and GBA, and TMS-derived parameters (Table 4).

An additional comparison was made between the same group of individuals with no production of SSADH protein to two other groups: the first including subjects in whom protein variants led to stability, folding, or oligomerization defects, and a second in which the resultant defect was catalytic because of affecting structural elements essential to catalysis or belonging to the NAD^+^ “sitting” groove. This comparison showed that with respect to individuals with a stability, folding, or oligomerization defect, those with no protein and a catalysis/NAD + binding defect had significantly lower total scores of their total CSS (*p* = 0.02) and CSS cognitive domain (*p* = 0.008), lower adaptive function test scores (*p* = 0.04) and more subjects with dyskinesia (*p* = 0.03). There was no difference between these groups in any other phenotype parameter assessed (Table 4).

**Figure 3:**
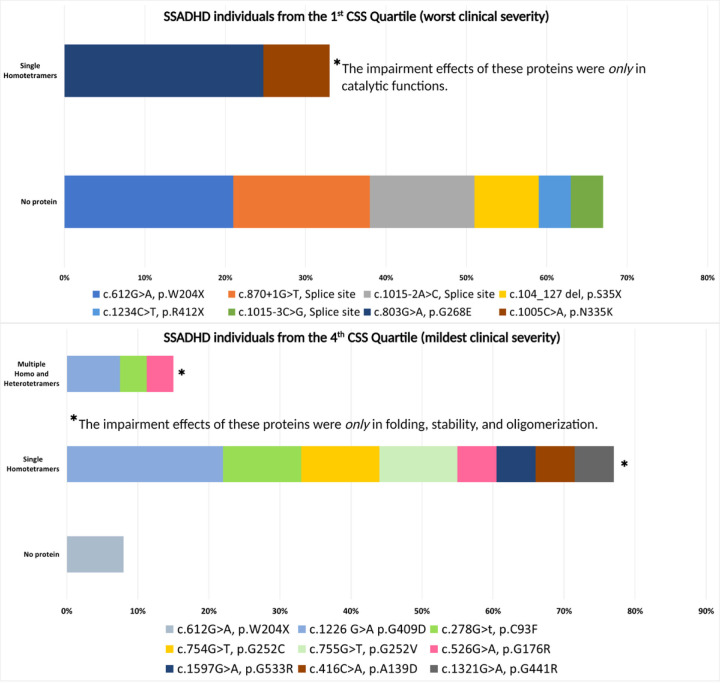
Comparison of the structural and functional impairment of SSADH proteins and their associated variants of SSADHD subjects in the 1st quartile (worst severity, above) vs. 4th quartile (mildest severity, below) of the SSADHD clinical severity score (CSS).

Division of the total CSSs of our study group to quartiles showed that 13 subjects were found in the top quartile (CSS ≥ 19.25), indicating the mildest clinical severity (patients #1, #5, #11, #12, #16, #23, #35, #36, #37, #47, #51, #53, #54). The vast majority (92%) of subjects from this subgroup had single homotetrameric or multiple homo or heterotetrameric SSADH proteins, all (100%) of which were affected by impaired stability, folding, or oligomerization. One subject (#1) from this subgroup had a combination of two nonsense variants presumed to synthesize only truncated SSADH polypeptide chains which cannot assemble the oligomeric SSADH, resulting in no functional SSADH protein produced. Out of the 18 missense variants of this subgroup, 6 (33%) were c.1226 G > A p.G409D, and 3 (17%) were c.278G > T, p.C93F (Fig. 3). Conversely, out of the 12 most severely affected subjects (#4, #14, #15, #18, #28, #29, #33, #34, #43, #44, #50, and #56) (all with CSS < 15, in the lower quartile), eight were predicted to have no protein being produced (by a combination of nonsense and splice-site variants), and four had single homotetrameric proteins, all (100%) which were impaired in catalysis abilities (three had one missense variant affecting catalysis that accompanied a nonsense variant and one had a splice site variant). There were no individuals in this subgroup with multiple homo or heterotetrameric proteins. Three out of the four (75%) missense variants of this subgroup were c.803G > A, p.G268E, and the other one was c.1005C > A, p.N335K (Fig. 3).

Compared to the rest of the study group, the 14 individuals with splice-site variants (12 of whom were compound heterozygotes) had significantly lower scores of the total CSS (mean ± SD of 15.5 ± 2.9 vs. 17.8 ± 2.5, *p* = 0.01) and CSS psychiatric domain (mean ± SD of 2.6 ± 1.2 vs. 3.4 ± 1.7, *p* = 0.04). These groups had no differences in other demographic, clinical, biochemical, neuroimaging, or neurophysiologic parameters. Interestingly, the single splice-site variant with Δ score < 0.5 was found in two participants with contrasting clinical courses. The first one (patient # 19), with a milder clinical outcome, had an additional missense variant (c.278G > T), resulting in milder impairments of the protein’s stability and folding. The second one (patient # 59), who had a severe clinical picture including drug-resistant seizures, had an additional non-sense mutation (c.1234C > T) resulting in a truncated protein.

## Discussion

SSADHD is a unique inherited disorder of GABA metabolism characterized by a particular phenotype that varies in severity. This study describes the first report of a genotype-phenotype correlation of SSADHD, performed on the largest studied cohort of individuals with this condition. Predictions of a genotype-phenotype correlation in monogenic diseases (e.g., phenylketonuria^34^) are typically performed by associating genetic variants to their frequency in alleles and genotypes and finally to phenotypes. In this study, in addition to the in silico analyses we performed on individual variants, we assessed the relationship between the SSADH protein population synthesized by each subject to the molecular effect derived from the combination of their variants. Our bioinformatic analyses revealed that *ALDH5A1* variants resulting in a truncated or lack of SSADH protein, as opposed to having single homotetramers or a mixed population homo and heterotetramers, are associated with worse clinical severity. Additionally, severe clinical outcomes in SSADHD coincided with impairment in the SSADH catalytic sites, as opposed to impairments in its folding, stability, or oligomerization. Considering SSADHD is an autosomal recessive inherited condition and SSADH is an oligomeric protein, knowledge of the *ALDH5A1* allelic variants is informative only if their resultant global molecular effect on the SSADH protein is elucidated. Hence, we propose that the genetic assessments of SSADHD individuals should be protein-focused.

This study’s findings determined that the 32 allelic variants (eight of which are novel), present in 41 unique allelic combinations (Table 1) in 58 SSADHD participants, were pathogenic. Considering the autosomal recessive inheritance of SSADHD, the consequences of these variants on the phenotype of the disease cannot be explained or attributed to a single allelic variant, irrespective of its type (missense, nonsense, frame-shift, or splice site). The complexity of the genotypic profile of our study population prompted us to perform an in-depth analysis of the effect of every single variant on SSADH protein structure and function and estimate the resultant effect of the combination of variants for each study participant. This innovative approach using extensive cross-examination between genotype, synthesized protein, and phenotype has yielded new correlations and may serve as a model for other rare diseases of similar inheritance.

The information gathered from our extensive variant analysis was used to predict disease presentation using the sizeable clinical database of our natural history study. Specifically, and in contrast with other studies which were limited in their assessment of the genotype-phenotype relationship by a lack of well-characterized phenotypical information^6, 7, 35^, the clinical phenotype of our patients was thoroughly characterized with a validated clinical severity score along with several quantitative clinical and neurometabolic parameters. This allowed us to more precisely assess the impact of the predicted protein number and functionality on disease outcomes.

A major outcome of the study is the finding that having variant combinations resulting in no SSADH protein or a truncated enzyme predicted worse overall clinical severity, worse cognitive abilities, worse psychiatric symptomatology, and increased seizure intensity. Additionally, we saw that variants resulting in multiple homo or heterotetrameric proteins were associated with fewer seizures and dyskinetic movement disorders than variants resulting in no protein or single homotetrameric proteins. This could be due to positive complementation effects resulting from the combination of polypeptide chains in the SSADH tetramer or from mRNA interallelic splicing that restores one healthy wild-type polypeptide chain. Comparison of the mutated protein structures and functions in subjects whose CSS fell in the 1st and 4th CSS quartiles further supported these observations. Most subjects from the 1st (worst severity) quartile had no protein, and none had multiple homo and heterotetrameric proteins. In contrast, only one subject from the 4th (mildest severity) CSS quartile had no protein. Why a lack of the SSADH enzyme coincides with the worst clinical outcome as compared to having single homotetrameric or multiple homo and heterotetrameric SSADH proteins can be intuitive, based on the fact that even malfunctioning enzyme variants can provide a minimum of catalytic activity. Moreover, the functional or partly functional SSADH protein has a tetrameric assembly. In compound heterozygous SSADHD subjects, this may lead to a possibility of many polypeptide chain combinations resulting in different clinical phenotypes. This phenomenon is also observed in other inherited metabolic disorders; in aromatic L-amino acid decarboxylase (AADC) deficiency, a splicing mutation leading to the absence of the enzyme is associated with the most severe clinical phenotype^36^. In phenylketonuria, splicing mutations are predicted to affect protein synthesis critically and worsen clinical outcomes. Further, in compound heterozygous phenylketonuria patients, variable production of phenylalanine and degrees of clinical severity depend on the combined effect of their two variants^37^.

As expected, the expression of ALDH5A1 was also lower in subjects who completely lacked the protein. Conceptually, it would be anticipated that a complete lack of the SSADH enzyme would result in higher values of cerebral and systemic GABA and its metabolites and accordingly, in cortical inhibition. However, no differences were found between protein subgroups in the age-adjusted means of MRS-derived GABA/NAA ratio, plasma GABA, GHB, and GBA levels, and TMS-derived indices of cortical inhibition. This could result from the small sample size of these subgroups or the absence of MRS and TMS data in the multiple homo and heterotetrameric proteins subgroup. It is also possible that this lack of correlation resulted from other multifactorial influences and complex GABAergic homeostatic mechanisms affecting the concentrations of GABA and its metabolites. GABA (and GABA-related metabolites) were shown to be dependent on GABA receptor expression (known to be downregulated in SSADHD)^38–40^ and polymorphisms in genes related to the GABA shunt^6^. Longitudinal measurement of these neurotransmitters will be needed to estimate the trajectory of their concentrations in relation to genotype.

Another significant outcome of the study is that subjects whose variants result in proteins impaired in stability, folding, or oligomerization have better overall clinical outcomes and adaptive functions than those with no protein or protein with impaired catalytic function. Here again, comparisons of subjects’ protein structural and functional profiles in the 1st and 4th quartiles of clinical severity scores were informative. The type of protein impairment observed in patients within the 1st CSS quartile (worst severity) was limited to impairment in catalytic function, as opposed to all subjects in the 4th quartile who only had single homotetrameric or homo and heterotetrameric proteins impaired in stability, folding, or oligomerization. In other inherited metabolic disorders, it has been reported that defects in folding, stability, or oligomerization are less disruptive than those resulting in loss of function from catalytic defects. In AADC deficiency, for example, purified recombinant pathogenic variants prone to misfolding or aggregation were shown to maintain catalytic activity, contributing to milder clinical phenotypes. In contrast, variants resulting in catalytic impairments usually lead to loss of function despite the lack of their structural disassembly^41^. Another example may be provided from phenylketonuria, in which it was demonstrated that residual activity of phenylalanine hydroxylase is the major determinant for disease severity in functionally hemizygous patients^42^. While folding and stability defects affect the oligomeric equilibrium between tetrameric and dimeric species of phenylalanine hydroxylase, they are related to milder forms of the disease^43^. The explanation for these findings could also stem from evidence demonstrating that the catalytic loop of the enzyme is influenced by environmental redox status, which in turn can lead to its structural modifications^5^. Two cysteine residues mapping on that catalytic loop regulate this process: Cys340 and Cys342. Amino acid substitutions altering the mobility of the 2-Cys loop may negatively affect the proper response to reactive oxygen species and change in redox status. Among the identified ALDH5A1 variants, N335K and G441R are in proximity to the 2-Cys loop and could be responsible for the worst phenotypes characterizing functionally hemizygous SSADHD subjects bearing them.

Our findings also have implications for gene replacement therapy for SSADHD. Since a functional SSADH is arranged in a tetrameric form, the proper assembly of the enzyme after gene therapy may ultimately govern therapeutic outcomes and efficacy. It is generally accepted that SSADH variant carriers are non-symptomatic. However, reports have suggested that non-pathogenic SSADH polymorphism leading to ~ 82% enzyme activity might suffice to contribute to cognitive decline and reduced survival in the elderly^44^. The knowledge gained from our structural and functional analyses of pathogenic *ALDH5A1* variants may thus be useful in the design of gene-editing or gene-replacement strategies, helping predict the functionality of the monomers produced by the wild-type gene when they assemble with the patients’ diseased monomers, and optimize the utility of gene therapy.

There are limitations to our study. While the genetic and protein analyses we performed are from the largest cohort of SSADHD patients ever studied, not all participants underwent all the neuroimaging, neurophysiologic, and neuropsychiatric assessments. This limitation is common in rare disease research, especially when affected individuals have a low tolerance to lengthy study procedures without sedation. Further, it must be pointed out that the bioinformatic predictions of SSADH structure and function are based on the enzyme’s crystal structure. Crystal structures are “frozen” models that cannot be used to predict the structural mobility of enzymes in their active state. In the case of SSADH, working with the crystal structure of the protein may have led to underestimating the impact of some variants on the catalytic capacity of the enzyme and disease presentation. Future studies will be needed to address this point, where recombinant SSADH variants will be cloned and expressed in appropriate cell models in combinations matching those of the individuals enrolled in our natural history study. These in vitro models would then be used to perform “personalized” predictions between the variant profile of each patient, enzyme kinetics parameters, and the patient’s clinical presentation. Lastly, as discussed above, other genetic factors likely contribute to disease presentation. These factors include the patient’s family genetic background, genes involved in the expression of GABA receptors, and receptors known to regulate downstream GABA signaling pathways. The expression of GABA receptors (their subunits) was not assayed in our study samples but should be included in customized profiler arrays in the future. Such information would complete the neurobiological interpretation of our findings related to the regulatory changes of GABA receptors in response to the hyperGABAergic state of the patients.

## Conclusions

This is the first comprehensive study of genotype-to-protein-to-phenotype correlations in SSADHD, an autosomal recessive inherited disorder with a unique neurometabolic phenotype. Bioinformatics and *in silico* modeling of a large number of *ALDH5A1* variants were used to predict the impact of the variants and variant combinations on protein structure and function. This information, coupled with the extensive clinical information gathered from the SSADHD natural history study, provided significant and novel insights into the relationships between gene, gene product, and disease phenotype. Worse clinical outcome was found in SSADHD subjects with a resultant lack of protein, as opposed to single homotetramers or multiple homo and heterotetramers. A milder clinical severity was seen in those whose resultant proteins were impaired in their stability, folding, or oligomerization as opposed to catalytic sites or lacking a protein. These findings are clinically relevant and potentially useful for prognostic estimations, disease management, and patient selection in future gene replacement therapy trials. Importantly, our approach to studying a genotype-phenotype relationship, including protein structure and function, may serve as a template to determine genotype-to-protein-to-phenotype relationships in other autosomal recessive rare disorders.

## Figures and Tables

**Table 1:** *In-Silico* analyses of the 32 *ALDH5A1* allelic variants found in SSADHD individuals of this study.

Nucleotide Variant	Protein Variant	Exon/Intron	Domain	SpliceAJ Dscore^1^	CONSURF	BJNDPROFX ΔΔG (Kcal/mol)	SIFT	POLYPHEN-2	CUPSAT Atom potential Torsion potential Mean ΔΔG (Kcal/mol)
c.612G > A	W204X	Exon 4	NAD^+^ binding						
c.1234C > T	R412X	Exon 8	Catalytic domain						
c.1015–2A > C	Splice site	Intron 7		0.99 (loss) 0.88 (gain)					
c.1597G > A	G533R	Exon 10	Oligomerization		6	0.511	0.01 deleterious	1.0 Probably damaging	Destabilizing Unfavorable −1.27
c.608C > T	P203L	Exon 3	NAD^+^ binding		8		0.00 deleterious	1.0 Probably damaging	Destabilizing Unfavorable −3.85
c.803G > A	G268E	Exon 5	NAD^+^ binding		7		0.00 deleterious	1.0 Probably damaging	Stabilizing Favorable 1.95
c.967_968dupCA	Q323HfsX3	Exon 6	Catalytic domain						
C.1226G > A	G409D	Exon 8	Catalytic domain		5		0.00 deleterious	1.0 Probably damaging	Destabilizing Favorable −0.1
c.1323dupG	P442AfsX18	Exon 8	Catalytic domain						
c.870 + 3delA	Splice site	Intron 5		0.75 (loss)					
c.526G > A	G176R	Exon 3	Oligomerization		2	0.000	0.00 deleterious	1.0 Probably damaging	Destabilizing Unfavorable −0.85
c.416C>A	A139D	Exon 2	NAD^+^ binding		7		0.01 deleterious	1.0 Probably damaging	Destabilizing Unfavorable −0.97
c.104_127 del	S35X	Exon 1	Mitochondrial targeting sequence						
c.517C>T	R173C	Exon 3	NAD^+^ binding		2		0.00 deleterious	1.0 Probably damaging	Stabilizing Favorable 0.58
c.1321G >A	G441R	Exon 8	Catalytic domain		8		0.00 deleterious	1.0 Probably damaging	Destabilizing Unfavorable −3.15
c.1402 + 2T >C	Splice site	Intron 9		0.97 (loss)					
c.278G >T	C93F	Exon 1	NAD^+^ binding		7		0.63 tolerated	0.999 Probably damaging	Stabilizing Unfavorable 2.16
c.1015–3C > G	Splice site	Intron 7		0.39 (loss) 0.40 (gain)					
c.754G >T	G252C	Exon 5	NAD^+^ binding		4		0.00 deleterious	1.0 Probably damaging	Destabilizing Unfavorable −8.12
c.621delC	S208VfsX3	Exon 4	NAD^+^ binding						
c.127delC	Q43SfsX47	Exon 1	Mitochondrial targeting sequence						
c.587G > A	G196D	Exon 3	NAD^+^ binding		9	0.00	deleterious	1.0 Probably damaging	Destabilizing Unfavorable −8.19
c.870 + 1G > T	Splice site	Intron 6		0.75 (loss)					
Deletion 6p22.3	No SSADH synthesis								
c.644_647delTGGG	V215GfsX11	Exon 4	NAD^+^ binding						
c.1558G > C	G520R	Exon 10	NAD^+^ binding		6	0.01	deleterious	1.0 Probably damaging	Destabilizing Favorable −2.17
c.755G > T	G252V	Exon 5			4	0.00	deleterious	1.0 Probably damaging	Destabilizing Unfavorable −7.04
c.755G > A	G252D	Exon 5			4	0.13	tolerated	1.0 Probably damaging	Destabilizing Unfavorable −4.48
c.1294A > C	M432L	Exon 8	Catalytic domain		5	0.09	tolerated	0.700 Possibly damaging	Destabilizing Unfavorable −0.67
c.610–2A > G	Splice site	Intron 4		0.96 (loss) 0.44 (gain)					
c.1005C > A	N335K	Exon 6	Catalytic domain		8	0.05	deleterious	0.999 Probably damaging	Destabilizing Unfavorable −1.01
c.1388del	D463VfsX2	Exon 9	Catalytic domain		8				

1. Hogema BM, Gupta M, Senephansiri H, et al. Pharmacologic rescue of lethal seizures in mice deficient in succinate semialdehyde dehydrogenase. *Nat Gen*

2. Akaboshi S, Hogema BM, Novelletto A, et al. Mutational spectrum of the succinate semialdehyde dehydrogenase (ALDH5A1) gene and functional analysis mutations in patients with SSADH deficiency. *Hum Mutat* 2003;22(6):442–450.

3. Kim YG, Lee S, Kwon OS, et al. Redox-switch modulation of human SSADH by dynamic catalytic loop. *EMBO J* 2009;28(7):959–968.

4. Attri SV, Singhi P, Wiwattanadittakul N, et al. Incidence and Geographic Distribution of Succinic Semialdehyde Dehydrogenase (SSADH) Deficiency. *JIMD re*

5. Chambliss KL, Caudle DL, Hinson DD, et al. Molecular cloning of the mature NAD(+)-dependent succinic semialdehyde dehydrogenase from rat and human homology, and tissue expression. *J Biol Chem* 1995;270(1):461–467.

6. DiBacco ML, Pop A, Salomons GS, et al. Novel ALDH5A1 variants and genotype: Phenotype correlation in SSADH deficiency. *Neurology* 2020;95(19):e2675

7. Pearl PL, Parviz M, Vogel K, et al. Inherited disorders of gamma-aminobutyric acid metabolism and advances in ALDH5A1 mutation identification. *Dev Med*

8. Pop A, Smith DEC, Kirby T, et al. Functional analysis of thirty-four suspected pathogenic missense variants in ALDH5A1 gene associated with succinic sem *Mol Genet Metab* 2020;130(3):172–178.

9. Jansen EE, Struys E, Jakobs C, et al. Neurotransmitter alterations in embryonic succinate semialdehyde dehydrogenase (SSADH) deficiency suggest a heig development. *BMC Dev Biol* 2008;8:112.

10. Yamakawa Y, Nakazawa T, Ishida A, et al. A boy with a severe phenotype of succinic semialdehyde dehydrogenase deficiency. *Brain Dev* 2012;34(2):107–

**Table 2:** Allelic variants, zygosity, resultant proteins combination, and eventual protein impairment effect of the SSADHD patients included in the study.

Patient	Allele_1	Allele_2	Zygosity	Protein variants combination	Effect
1	c.612G > A	c.612G > A	Homozygosis	Truncated protein	No protein
2	c.612G > A	c.1234C > T	Compound heterozygosis	Truncated proteins	No protein
3	c.612G > A	c.1234C > T	Compound heterozygosis	Truncated proteins	No protein
4	c.612G > A	c.1015–2A > C	Compound heterozygosis	Truncated protein/impaired splicing	No protein
5	c.1015–2A > C	c.1597G > A	Compound heterozygosis	Hemizygote G533R homotetramers	Oligomerization
6	c.608C > T	c.608C > T	Homozygosis	P203L homotetramer	Catalysis
7	c.612G > A	c.803G > A	Compound heterozygosis	Hemizygote G268E homotetramers	Catalysis
8	c.967_968dupCA	c.1597G > A	Compound heterozygosis	Hemizygote G533R homotetramers	Oligomerization
9	c.1226G > A	c.1323dupG	Compound heterozygosis	Hemizygote G409D homotetramers	Stability/folding
10	c.612G > A	c.870 + 3delA	Compound heterozygosis	Truncated protein/impaired splicing	No protein
11	c.526G > A	c.1226G > A	Compound heterozygosis	Mixed protein population of G409D and G176E homotetramers and heterotetramers	Folding/stability and oligomerization
12	c.1015–2A > C	c.416C > A	Compound heterozygosis	Hemizygote A139D homotetramers	Stability/folding
13	c.517C > T	c.1015–2A > C	Compound heterozygosis	Hemizygote R173C homotetramers	Oligomerization
14	c.104_127 del	c.1015–2A > C	Compound heterozygosis	Truncated protein/impaired splicing	No protein
15	c.104_127 del	c.1015–2A > C	Compound heterozygosis	Truncated protein/impaired splicing	No protein
16	c.612G > A	c.1321G > A	Compound heterozygosis	Hemizygote G441R homotetramers	Stability/folding
17	c.612G > A	c.1402 + 2T > C	Compound heterozygosis	Truncated protein/impaired splicing	No protein
18	c.612G > A	c.803G > A	Compound heterozygosis	Hemizygote G268E homotetramers	Catalysis
19	c.278G > T	c.1015–3C > G	Compound heterozygosis	Hemizygote C93F homotetramers	Stability/folding
20	c.612G > A	c.803G > A	Compound heterozygosis	Hemizygote G268E homotetramers	Catalysis
21	c.1226G > A	c.1226G > A	Homozygosis*	G409D homotetramers	Stability/folding
22	c.754G > T	c.754G > T	Homozygosis*	G252C homotetramers	Stability/folding
23	c.754G > T	c.754G > T	Homozygosis*	G252C homotetramers	Stability/folding
24	c.754G > T	c.754G > T	Homozygosis*	G252C homotetramers	Stability/folding
25	c.1226G > A	c.1226G > A	Homozygosis*	G409D homotetramers	Stability/folding
26	c.612G > A	c.1597G > A	Compound heterozygosis	Hemizygote G533R homotetramers	Oligomerization
27	c.612G > A	c.803G > A	Compound heterozygosis	Hemizygote G268E homotetramers	Catalysis
28	c.612G > A	c.803G > A	Compound heterozygosis	Hemizygote G268E homotetramers	Catalysis
29	c.621delC	c.621delC	Homozygosis*	Truncated/inactive	No protein
30	c.127delC	c.803G > A	Compound heterozygosis	Hemizygote G268E homotetramers	Catalysis
31	c.1234C > T	c.1234C > T	Homozygosis	Truncated/inactive	No protein
32	c.870 + 1G > T	c.870 + 1G > T	Homozygosis*	Truncated/inactive	No protein
33	c.870 + 1G > T	c.870 + 1G > T	Homozygosis*	Truncated/inactive	No protein
34	c.278G > T	c.621delC	Compound heterozygosis	Hemizygote C93F homotetramers	Stability/folding
35	c.278G > T	c.621delC	Compound heterozygosis	Hemizygote C93F homotetramers	Stability/folding
36	c.526G > A	Deletion 6p22.3	Compound heterozygosis	Hemizygote G176E homotetramers	Folding/oligomerization
37	c.278C > T	c.803G > A	Compound heterozygosis	Mixed protein population of C93F and G268E homotetramers and heterotetramers	Catalysis**
38	c.278G > T	c.278G > T	Homozygosis	C93F homotetramers	Stability/folding
39	c.587G > A	c.587G > A	Homozygosis	G196D homotetramers	Stability/folding
40	c.644_647delTGGG	c.644_647delTGGG	Homozygosis	Truncated/inactive	No protein
41	c.1226G > A	c.1558G > C	Compound heterozygosis	Mixed protein population of G409D and G520R homotetramers and heterotetramers	Stability/folding and oligomerization
42	c.612G > A	c.612G > A	Homozygosis	Truncated protein	No protein
43	c.612G > A	c.612G > A	Homozygosis	Truncated protein	No protein
44	c608C > T	c608C >T	Homozygosis	P203L homotetramer	Catalysis
45	c.1226G > A	c.1226G > A	Homozygosis*	G409D homotetramers	Stability/folding
46	c.755G > T	c.755G > T	Homozygosis	G252V homotetramers	Stability/folding
47	c.755G > A	c.1226G > A	Compound heterozygosis	Mixed protein population of G252D and G409D homotetramers and heterotetramers	Stability/folding
48	c.610–2A > G	c.1294A > C	Compound heterozygosis	Hemizygote M432L homotetramers	Catalysis
49	c.1005C > A	c.1015–2A > C	Compound heterozygosis	Hemizygote N335K homotetramers	Catalysis
50	c.1226G > A	c.278G > T	Compound heterozygosis	Mixed protein population of C93F and G409D homotetramers and heterotetramers	Stability/folding
51	c.1226G > A	c.1226G > A	Homozygosis	G409D homotetramers	Stability/folding
52	c.1226G > A	c.1226G > A	Homozygosis	G409D homotetramers	Stability/folding
53	c.1226G > A	c.1226G > A	Homozygosis	G409D homotetramers	Stability/folding
54	c.1226G > A	c.1226G > A	Homozygosis	G409D homotetramers	Stability/folding
55	c.1234C > T	c.1015–3C > G	Compound heterozygosis	Truncated protein/impaired splicing	No protein
56	c.803G > A	c.1558G > C	Compound heterozygosis	Mixed protein population of G268E and G520R homotetramers and heterotetramers	Catalysis**
57	c.803G > A	c.1558G > C	Compound heterozygosis	Mixed protein population of G268E and G520R homotetramers and heterotetramers	Catalysis**
58	c.1388del	c.803G > A	Compound heterozygosis	Hemizygote G268E homotetramers	Catalysis

* Consanguinity.

** Also a minor effect in stability/folding

**Table 3:** In silico evaluation of SSADHD-related variants’ steric hindrance resultant from substituted neighboring residues within the monomeric structure of SSADH.

Variant	Steric Hindrance
G252V	67.95
G252C	56.81
G409D	56.27
G441R	55.84
G252D	55.70
C93F	41.87
P203L	41.12
G520R	38.18
G196D	33.42
G533R	31.74
G268E	29.06
R173C	25.53
A139D	21.94
M432L	15.31
N335K	14.55
G176R	11.03

**Table 4:** Relationship between genotype expressed in clusters of protein quantity and impairment effect to clinical phenotype. Individuals with no SSADH protein are compared to A) those with Single Homotetramers and Multiple Homo and Heterotetramers and B) those with different effects of protein impairments.

		Quantitative Protein Groups N = 58 (%)			Impairment Effect Groups N = 58 (%)		
Phenotype features	No Protein* N = 16(28)	Single Homotetramers N = 35(60)	Multiple Homo and Heterotetramers N = 7(12)	p**	Folding/Stability/Oligomerization N = 29 (50)	Catalysis N = 13 (22)	P***
**Age**,							
years, median (IQR)	9.8 (6.9–21.5)	10.4 (5.3–15.0)	7.8 (4.2–11.1)	*0.22*	9.6 (4.2–14.2)	11.1 (8.0–14.4)	*0.33*
**Sex**							
Male/Female	9 (56)/7 (44)	16 (46)/19 (54)	4 (57)/3 (43)	*0.72*	4 (31)/9 (69)	16 (55)/13 (45)	*0.28*
**Consanguinity**	3 (19)	6 (17)	0 (0)	*0.45*	6 (21) 0 (0)		*0.19*
**Gene expression**, 2^^^ΔCT							
*ALDH5A1* (N = 23)	0.01 ± 0.005 (N = 8)	0.02 ± 0.01 (N = 14)	0.05 ± 0.0 (N = 1)	**0.001**	0.02 ± 0.01 (N = 11)	0.01 ± 0.008 (N = 4)	*0.07*
*Abat* (N = 23)	0.03 ± 0.03 (N = 8)	0.02 ± 0.007 (N = 14)	0.02 ± 0.0 (N = 1)	*0.43*	0.02 ± 0.007 (N = 11)	0.02 ± 0.009 (N = 4)	*0.43*
*GLS* (N = 23)	0.11 ± 0.04 (N = 8)	0.09 ± 0.02 (N = 14)	0.11 ± 0.0 (N = 1)	*0.46*	0.09 ± 0.09 (N = 11)	0.09 ± 0.004 (N = 4)	0.51
**Clinical Severity Score (CSS)**							
Total score	15.5 ± 2.9	17.8 ± 2.6	18.5 ± 1.3	**0.008**	18.7 ± 1.9	16.2 ± 2.6	**0.02**
Cognitive	1.9 ± 0.8	2.6 ± 0.9	2.8 ± 0.3	**0.01**	2.8 ± 0.9	2.4 ± 0.7	**0.008**
Communication	2.5 ± 0.6	2.8 ± 0.9	3.1 ± 0.9	*0.27*	2.9 ± 0.9	3.0 ± 1.0	*0.33*
Motor	3.7 ± 1.0	3.7 ± 0.8	3.2 ± 0.7	*0.47*	3.7 ± 0.7	3.3 ± 0.9	*0.42*
Psychiatry	2.6 ± 1.3	3.4 ± 1.1	4.0 ± 0.8	**0.04**	3.6 ± 1.1	3.2 ± 1.0	*0.10*
Epilepsy	3.6 ± 1.2	4.1 ± 1.2	5.0 ± 0.0	**0.04**	4.5 ± 1.0	3.7 ± 1.2	*0.10*
**Neuropsychologic assessments**							
FSIQ (N = 27)	47.8 ± 13.1 (N = 8)	52.1 ± 13.1 (N = 18)	57.0 ± 0.0 (N = 3)	*0.67*	53.3 ± 13.4 (N = 14)	49.6 ± 11.6 (N = 5)	*0.62*
Adaptive composite score (N = 33)	56.5 ± 11.2 (N = 9)	61.4 ± 15.1 (N = 23)	75.0 ± 0.0 (N = 3)	*0.41*	66.6 ± 12.9 (N = 16)	52.8 ± 15.7 (N = 8)	**0.04**
ASD assessed by ADOS-2 (N = 30)	6 (67) (N = 9)	11 (52) (N = 21)	-	*0.37*	6 (43) (N = 14)	5 (71) (N = 7)	*0.35*
**Movement disorders**							
Ataxia	11 (69)	17 (49)	2 (29)	*0.17*	8 (61)	11 (38)	*0.10*
Dyskinesia	7 (44)	7 (20)	0 (0)	**0.05**	3 (10)	4 (31)	**0.03**
Dystonia	2 (12)	5 (14)	2 (29)	*0.58*	3 (10)	4 (31)	*0.22*
**Epilepsy**							
Seizures	9 (56)	21 (60)	0 (0)	**0.01**	13 (45)	8 (61)	*0.55*
Drug-resistant seizures	3 (43)	6 (29)	-	*0.48*	2 (15)	4 (50)	*0.20*
EEG abnormalities	15 (94)	30 (86)	2 (29)	**< 0.001**	21 (72)	11 (85)	*0.31*
EEG- diffuse background slowing	9 (56)	17 (49)	1 (14)	*0.16*	13 (45)	4 (31)	*0.39*
EEG- epileptiform activity	5 (31)	12 (34)	1 (14)	*0.58*	6 (20)	7 (54)	*0.10*
**MRS GABA/NAA**, EMM (SE)							
Basal ganglia (N = 13)	0.19 (0.02) (N = 3)	0.20 (0.01) (N = 9)	-	*0.56*	0.19 (0.01) (N = 7)	0.22 (0.02) (N = 2)	*0.62*
Posterior cingulate gyrus (N = 19)	0.21 (0.01) (N = 7)	0.22 (0.01) (N = 11)	-	*0.46*	0.21 (0.01) (N = 8)	0.23 (0.01) (N = 3)	*0.42*
Occipital cortex (N = 12)	0.16 (0.01) (N = 7)	0.17 (0.02) (N = 5)	-	*0.62*	0.16 (0.02) (N = 3)	0.19 (0.03) (N = 2)	*0.74*
**Biochemical metrics**, EMM (SE)							
GABA, μ/L (N = 43)	3.1 (0.2) (N = 16)	2.8 (0.2) (N = 24)	3.0 (0.6) (N = 3)	*0.70*	2.9 (0.2) (N = 20)	2.5 (0.4) (N = 7)	*0. 52*
GHB, μ/L (N = 34)	274.2 (224.4) (N = 11)	467.5 (154.5) (N = 22)	1009.6 (732.9) (N = 1)	*0. 59*	580.2 (178.1) (N = 16)	243.1 (290.9) (N = 7)	*0.47*
GBA, μ/L (N = 29)	0.09 (0.009) (N = 9)	0.07 (0.006) (N = 18)	0.08 (0.018) (N = 2)	*0.20*	0.07 (0.006) (N = 16)	0.07 (0.01) (N = 4)	*0.21*
**TMS**, EMM (SE)							
rMT, %MO (N = 23)	72.2 (6.4) (N = 8)	62.9 (4.6) (N = 15)	-	*0.26*	66.0 (5.4) (N = 12)	54.9 (8.8) (N = 3)	*0.31*
CSR ms (N = 21)	221.7 (16.7) (N = 6)	198.4 (12.4) (N = 15)	-	*0.39*	175.6 (12.6) (N = 11)	241.9 (17.3) (N = 4)	*0.10*
LICI, log (MEPt/MEPc) (N = 18)	−0.02 (0.27) (N = 6)	−0.01 (0.18) (N = 12)	-	*0.97*	−0.03 (0.22) (N = 10)	0.24 (0.37) (N = 2)	*0.99*

ADOS-2- Autism Diagnostic Observation Schedule-Second Edition; ASD- Autism Spectrum Disorder; CSP- cortical silent period; EEG- Electroencephalogram; EEM- estimated marginal means adjusted for age; FSIQ- full scale intellectual quotient; GABA- γ-aminobutyrate; GHB- γ-hydroxybutyrate; GBA-guanidinobutyrate; IQR- Interquartile ratio; LICI- long interval intracortical inhibition; MEPc- motor evoked potential, condition; MEPt- motor evoked potential, test; MO- machine output; MRS- Magnetic resonance spectroscopy; ms- milliseconds; NAA- N-acetyl aspartate; NAD- SD- Standard deviation; SE- standard error; SSADHD- succinic semialdehyde dehydrogenase deficiency; TMS- transcranial magnetic stimulation; **Bold** indicates significant.

* The ‘No Protein’ group participates in two comparisons presented in this table: 1) with the ‘Single Homotetramers’ and ‘Multiple Homo and Heterotetramers’ groups; 2) with the two “Effect in impairment” groups.

** These p values represent the comparison between the groups ‘No protein,’ ‘Single Homotetramers,’ and ‘Multiple Homo and Heterotetramers.’

** These p values represent the comparison between the groups ‘No Protein,’ ‘Impairment effect in Folding/Stability/Oligomerization,’ and ‘Impairment Effect in Catalysis.’

## Data Availability

The datasets generated during and/or analyzed during the current study are available from the corresponding author on reasonable request.
